# Using Stochastic Simulation‐Estimation and Automated Model Development to Assess Power and Accuracy for Covariate Identification

**DOI:** 10.1002/psp4.70299

**Published:** 2026-07-21

**Authors:** Ya‐Han Hsu, Bárbara Costa, Nuno Vale, Thomas P. C. Dorlo, Mats O. Karlsson

**Affiliations:** ^1^ Department of Pharmacy Uppsala University Uppsala Sweden; ^2^ PerMed Research Group, RISE‐Health, Faculty of Medicine University of Porto Porto Portugal; ^3^ RISE‐Health, Department of Community Medicine, Health Information and Decision (MEDCIDS), Faculty of Medicine University of Porto Porto Portugal; ^4^ Laboratory of Personalized Medicine, Department of Community Medicine, Health Information and Decision (MEDCIDS), Faculty of Medicine University of Porto Porto Portugal; ^5^ Centre for Parasite Biology and Immunology, Department of Infectious Diseases National Health Institute Dr. Ricardo Jorge Lisbon Portugal

**Keywords:** automated model development (AMD), covariate identification, model building, power analysis, stochastic simulation and estimation (SSE), study design evaluation

## Abstract

When study designs are evaluated using clinical trial simulations for their ability to identify covariate effects in population pharmacokinetic (PopPK) modeling, it is typically assumed that the true model will be known at the data analysis stage. In this study, this was compared with the more realistic assumption that the PopPK model needs to be built on the data generated by the planned study. Three approaches were compared: (i) stochastic simulation and re‐estimation (SSE) with the simulation model, (ii) automated model development (AMD) with exploratory covariate search (AMD‐exploratory), and (iii) AMD forcing the covariate effect into the model from the start and reevaluating it in the end (AMD‐structural). With a simulated covariate effect (a hypothetical pregnancy effect on clearance), we assessed (i) the type 1 error (T1E) and the power of covariate identification and (ii) covariate parameter accuracy. The T1E rate was controlled in SSE and AMD‐exploratory but 20% inflated for AMD‐structural. The power of covariate identification in rich, medium, and sparse designs was (i) 99%, 100%, and 79% in SSE, (ii) 74%, 72%, and 41% in AMD‐exploratory, and (iii) 92%, 93%, and 80% in AMD‐structural. Sparse designs amplified power differences between strategies, with AMD‐exploratory often selecting alternative or no covariates. The rRMSE of covariate parameter estimates was lowest in SSE (27%, 22%, and 42%), followed by AMD‐exploratory (34%, 26%, and 49%) and then AMD‐structural (42%, 47%, and 60%). SSE provides optimistic power estimates as model building is data‐driven, while AMD‐based approaches incorporate model uncertainty and reflect real‐world analysis conditions.

## Study Highlights

- What is the current knowledge on the topic?
  ○ Stochastic simulation and estimation (SSE) is the standard approach for evaluating study design and covariate power in popPK, but it does not account for model‐building uncertainty, potentially overestimating power.
- What question did this study address?
  ○ We investigated how different approaches: SSE, automated model development with exploratory covariate search (AMD‐exploratory), and AMD with structural covariate forcing (AMD‐structural) affect type I error, power, and parameter accuracy in covariate identification.
- What does this study add to our knowledge?
  ○ We showed how different AMD approaches yield more realistic, though variable, outcomes by incorporating model‐building uncertainty compared to an SSE approach. Additionally, forcing covariates increased power but inflated type I error.
- How might this change drug discovery, development, and/or therapeutics?
  ○ Accounting for model‐building uncertainty in power estimation can improve study design, enabling more realistic covariate identification and better‐informed decisions in drug development.

## 1 Introduction

Clinical trial simulations are widely used in planning studies designed to evaluate drug therapies [1]. Population pharmacokinetic (PopPK) and pharmacodynamic (PopPD) models are typically used when longitudinal data are to be simulated [2]. In some situations, PopPK/PD models will also be used to analyze the actual trial results. When that is the case, it is of interest to investigate whether the informativeness of the trial design is sufficient for the intended purpose(s) of the study [3]. The most basic assessment is whether the simulation model parameters can be estimated with acceptable accuracy from the resulting study data [4]. When it comes to investigating the significance of a treatment or covariate effect in the planned trial (to evaluate if it is detected or not), the strategy is to first simulate data with the treatment/covariate effect included in the simulation model and secondly to analyze the resulting data with two models: one including (full model) and one excluding (reduced model) the treatment/covariate effect [5, 6]. Subsequently, the difference in goodness of fit, quantified as minus two times the log likelihood (−2LL), over many simulated trials forms the basis for concluding whether the risk of failing to identify an existing treatment/covariate effect (Type II error) is sufficiently low [7, 8].

Thus, the stochastic simulation and estimation (SSE), which is used to assure sufficiently informative study designs, assumes the PopPK/PD analysis of the real study data will not involve model building and will instead use the ideal model for the analysis. This is in stark contrast to how PopPK/PD analyses on real clinical trial data are performed [9]. There is often considerable model building, assessing alternative models regarding structure, covariates, and unexplained parameter and residual variability [10]. While modelers have been aware of this mismatch between the design and analysis, it has historically been infeasible to perform model building on each simulated data set of an SSE, as such model building has been manual.

Recent advancements in automated tools for popPK model building provide alternative strategies for treatment/covariate identification with model building [11, 12]. Pharmpy's automated model development (AMD) tool evaluates multiple model components and applies search algorithms automatically to perform model selection [11, 13]. This approach enables efficient model building, evaluation, and hypothesis testing regarding covariates [14, 15]. While developed to perform model building on real clinical data, it can also be used at the design stage to perform clinical trial simulations that are mimicking the analysis process.

**FIGURE 1:**
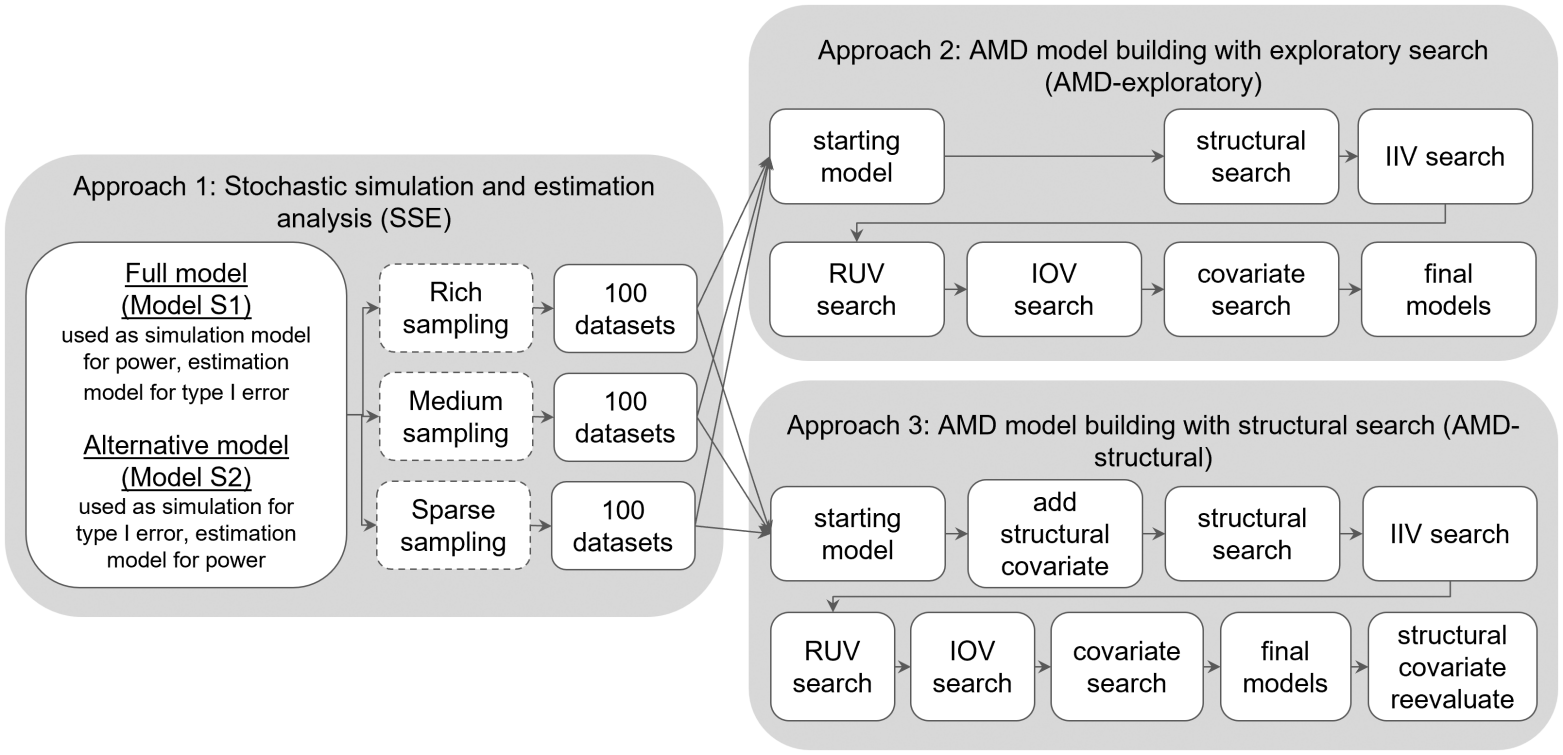
The workflow illustrates the assessment of covariate effects using SSE and AMD across different sampling designs. In Approach 1 (SSE), datasets of varying richness are simulated from the full model (Model S1, including the covariate effect; described below and estimated with an alternative model Model S2, excluding the covariate effect; described below) in power estimation; conversely, Model S2 was for simulation and Model S1 for estimation in type I error estimation. Approach 2 (AMD‐exploratory) and 3 (AMD‐structural) apply the AMD tool with two covariate model building strategies: An exploratory search, in which covariates are added through stepwise selection, and a structural search, in which the covariate is forced into the model and subsequently reevaluated, respectively.

In this study, we compared three approaches to identify power and the impact of study design on covariate selection: (i) SSE—simulating 100 datasets and estimating them using the same simulation model, (ii) AMD with exploratory covariate search (AMD‐exploratory)—incorporating the covariate effect in the model only if identified as significant, and (iii) AMD with structural covariate search (AMD‐structural)—forcing a specific covariate effect of interest into the model from the start and reevaluating it at the end (Figure 1). By comparing SSE with AMD, we assessed the robustness of power and parameter estimation while illustrating how model performance and covariate identification are impacted by study design and covariate modeling approaches.

## 2 Methods

### 2.1 Simulation Model and Designs

A one‐compartment model with first‐order absorption and first‐order elimination, including pregnancy as a covariate on clearance, was used as the structural basis for the simulation full model (Model S1). Table 1 presents the parameter estimates and variance components of the model. This model structure and parameter values were informed by the published PopPK model of dolutegravir (DTG) using the DolPHIN‐1 trial [16] and further bioavailability considerations from Kawuma et al. [17]. Model S1 was simplified and harmonized for simulation purposes rather than implemented as an exact reproduction on either model. In the reduced model (Model S2), the pregnancy effect on clearance (TH_CLpreg_) was fixed to 0 to represent the condition of no covariate effect for type I and type II error evaluation.

**TABLE 1:** Parameter values of the simulation model (Model S1). The full model code can be found in the supporting Information.

Parameter	Value	Description
Fixed Effects		
CL (TH_CL_)	1.5	clearance (L/h)
V	25	volume of distribution (L)
k_a_	0.75	absorption rate constant (1/h)
Pregnancy (TH_CLpreg_)	0.25	pregnancy effect [TVCL = TH_CL_*(1 + TH_CLpreg_)]
Variability parameters		
IIV for CL/F	25.5	inter‐individual variability for clearance (%)
Covariance for CL/F‐V/F	16.7	variance–covariance of CL and V (%)
IIV for V/F	22.4	inter‐individual variability for volume (%)
IIV for KA	44.7	inter‐individual variability for KA (%)
IOV for F	44.7	inter‐occasion variability for bioavailability, same in the pregnancy (occasion = 1) and postpartum (occasion = 2) period (%)
Proportional Error	0.35	proportional residual error (%)

A virtual population of 28 individuals was generated. Age and weight were simulated from uniform distributions using the NONMEM $SIMULATION command: age ranging from 19 to 42 years old and weight ranging from 44 to 160 kg. These covariates were included as candidate continuous predictors in the AMD covariate search (Table 2) to enable evaluation of covariate competition with the pregnancy effect. Individuals were simulated under a once‐daily dosing regimen of 50 mg at steady‐state. Pregnancy and postpartum were presented as two occasions within each individual. Each occasion represents an independent steady‐state condition for the design of within‐subject variation to study pregnancy‐related changes in drug PK. No explicit washout period was included between occasions. Inter‐occasion variability (IOV) was incorporated on bioavailability to account for within‐subject differences between pregnancy and postpartum. Sampling times were simulated at fixed time points (at pre‐dose and 0.5, 1, 2, 3, 4, 6, 8, and 24 h after treatment) in both the pregnancy and postpartum states, consistent with the design of the reference studies used to inform the simulation framework.

**TABLE 2:** Search space, search strategy and selection criteria in the AMD tool.

Module	Search space	Search algorithm	Selection criteria
Model search	absorption: first‐order (FO), zero order (ZO), sequential ZO‐FO; absorption delay: with or without lag time, 0/1/3 transit compartment with or without depot compartment; distribution: 1‐compartment, 2‐compartment; elimination: FO	reduced exhaustive stepwise algorithm; all models run with IIV structure [CL,V] + [MAT] (IIV on CL, V, MAT, and a covariance between IIV on CL and V) in this step	Bayesian Information Criterion (BIC) for mixed effect models^19^
IIV search	eta added on all PK parameters and all covariances included	two‐step top‐down exhaustive search	BIC for random effect models
RUV search	proportional, combined proportional and additive, power, IIV on RUV, time‐varying error	stepwise algorithm with Resmod approach	Likelihood ratio test (LRT) with *p*‐value 1%
IOV search	eta added on parameters with existing IIV	two‐step top‐down exhaustive search, same variance–covariance matrix as IIV	BIC for random effect models
Covariate search	categorical: pregnancy; continuous (linear or exponential): age, body weight	(1) AMD‐exploratory: all covariates tested with the forward‐inclusive stepwise covariate modeling (SCM) procedure (2) AMD‐structural: predefined covariate added prior to model search without testing, other covariates tested with forward‐inclusive SCM	Applied the same threshold, LRT with *p*‐value 5%: (1) AMD‐exploratory—for screening; (2) AMD‐structural—for re‐evaluation of predefined covariate

To investigate how data richness influences different approaches in identifying and assessing covariate effects, two additional sampling designs with varying levels of data sparsity were generated (100 datasets were generated for each sampling scenario). The medium design, which was mentioned previously, was used as the baseline in our study. The rich design extended this with additional samples at 12 and 18 h to provide more data in the elimination phase. For comparison, the sampling time of the sparse design was set at 2, 4, 8, and 24 h after treatment.

### 2.2 Stochastic Simulation Estimation (SSE) Scenarios

In the SSE analysis (Approach 1 in Figure 1) for identifying the pregnancy effect on clearance (CL‐PREG), the type I error (T1E) rate indicates the false‐positive rate of concluding CL‐PREG as a significant covariate when it's not, while the power indicates the ability of the covariate selection approach to identify the true CL‐PREG effect. In this study, the T1E rate and power of identifying CL‐PREG were evaluated at a 5% significance level using the likelihood ratio test (LRT), based on the differences in objective function values (dOFV) between the full model (Model S1, incorporating a 25% effect of pregnancy on clearance) and the reduced model (Model S2, excluding this covariate effect) among 100 simulated datasets. When estimating T1E, the reduced model (Model S2, without CL‐PREG) was used for data simulation, and the T1E rate was based on the percentage of reduced models in which CL‐PREG false‐positively showed statistical significance. Power, by reversing the simulation with the full model (Model S1, with CL‐PREG), was estimated based on the percentage of full models in which CL‐PREG was statistically significant.

SSE was performed with Perl‐speaks‐NONMEM (PsN, version 5.3.0; NONMEM version 7.5). PsN commands are provided in the supplemental material. The parametric power estimation (PPE) approach implemented in PsN was used to further map the relationship between sample size and statistical power for detecting the covariate effect [18]. The PPE results, shown only in the supporting Information, served as a reference, providing the complete SSE outcome instead of a direct comparison with the three approaches in this study.

### 2.3 Model Development With the AMD Tool

By using the AMD tool in Pharmpy (version 1.3.0, via the R package ‘pharmr’), new models for each SSE‐simulated dataset were developed. The settings and scope of AMD used in this study followed the previously published article, and the details are described in the following paragraphs [11]. Model estimations were performed in NONMEM (version 7.5) by the first‐order conditional estimation with interaction (FOCEI).

The starting model in AMD was a one‐compartment model with first‐order absorption and first‐order elimination, including proportional residual unexplained variability (RUV) with IIV on clearance (CL), volume of distribution (V), mean absorption time (MAT), and a correlation between CL and V. Initial estimates were set to 1.5 L/h for CL, 25 L for V, and 0.75 h for MAT. The search strategy in the AMD tool followed a sequential order of structural model, IIV, RUV, IOV, and covariates. The search space, searching algorithm, and the selection criteria in each module are listed in Table 2. Search space and search algorithm in the AMD tool define what and how model components were tested, and selection criteria determine how candidate models were ranked and selected. The Bayesian Information Criterion (BIC) for mixed effect models used as selection criteria in multiple steps is calculated based on OFV and contains penalties related to the number of estimated parameters [19].

Specifically, two covariate model building strategies, the AMD‐exploratory (approach 2 in Figure 1) and the AMD‐structural (approach 3 in Figure 1), were compared and evaluated in this study. Both approaches applied the forward‐inclusive stepwise covariate modeling (SCM) method with a 5% significance level. In AMD‐structural, the structural CL‐PREG was reevaluated with a LRT at a significance level of 5%. T1E and power were estimated as described in the SSE section (approach 1 in Figure 1). The main difference was that in approach 1, the same pair of full‐reduced models was applied for all 100 datasets; whereas approaches 2 and 3 took into account the model building process (exploratory or structured, as defined for each approach) by building different models across 100 datasets.

Although continuous covariates (age and body weight) were included in the AMD covariate search space and tested against pregnancy effect during model selection, pregnancy was selected as the primary covariate because: (i) its effect was directly parameterised in the simulation model using a known true effect size from DolPHIN‐1; and (ii) its binary nature avoids additional uncertainty form for continuous covariates, isolating the methodological comparison. No continuous covariate effect was simulated as the true relationship, so extrapolation of these findings to continuous covariates should be made with caution.

### 2.4 Covariate Identification and Comparison

We compared the three approaches (SSE, AMD‐structural, and AMD‐exploratory) in terms of their (1) T1E and power of covariate identification (described earlier), (2) bias, precision, and accuracy of coefficient estimation, and (3) goodness‐of‐fit, quantified using a complex‐adjusted model selection criterion. Robustness across varying data richness (sampling designs) was also evaluated.

The evaluation of the bias, precision, and accuracy of parameter estimates included only models with TH_CLpreg_. The bias and precision were assessed using the deviation of the mean from the true parameter value and the standard deviation (SD) of estimated parameter values, respectively. Accuracy was assessed using the root mean square error (RMSE), calculated as the square root of the mean squared difference between estimated and true values across simulations. To allow comparison across parameters of different magnitudes, the relative RMSE (rRMSE) was also calculated by dividing RMSE by the true value.

Goodness‐of‐fit between AMD‐built models and the full SSE model was compared using the differences in Bayesian Information Criterion (dBIC), calculated as dBIC = BIC_AMD_—BIC_full_. The BIC_AMD_ is the BIC of AMD‐built models and BIC_full_ is the BIC of the full SSE model (Model S1). Negative dBIC values indicate that the AMD‐built model has a lower, complexity‐adjusted BIC than the full SSE model, reflecting a realistic popPK scenario in trial data analysis. Conversely, positive dBIC values are attributed to the insufficient information in the simulated datasets and uncertainties during stepwise model building.

## 3 Results

### 3.1 Type 1 Error Rate and Power of Covariate Identification

We compared the three approaches (SSE, AMD‐structural, and AMD‐exploratory) in terms of T1E, power, parameter estimation, and goodness‐of‐fit across 100 simulated datasets for each sampling design. The T1E rate was inflated for the AMD‐structural approach, while controlled in both SSE and AMD‐exploratory (Table 3, footnote a). With a 5% significance level, the T1E rate in AMD‐structural was 17%, 18%, and 20% in the rich, medium, and sparse designs, respectively. The inflated T1E reveals the potentially increased risk of false‐positively identifying covariate effects with this model building approach. In SSE and AMD‐exploratory, T1E was 4%–5% in the rich and medium designs, while slightly increasing to 6%–7% in the sparse sampling designs.

**TABLE 3:** Identifiability and parameter estimates of pregnancy effect on clearance (CL‐PREG).

Design	Approach for identifying covariate effects	CL‐PREG identifiability		TH_CLpreg_ ^a^	
		Power	Type 1 error	Mean ± SD	RMSE (rRMSE%)
Rich	SSE	99%	5%	0.26 ± 0.07	0.07 (27%)
	AMD‐exploratory	74%	4%	0.28 ± 0.09	0.09 (34%)
	AMD‐structural	92%	17%	0.26 ± 0.11	0.11 (42%)
Medium	SSE	100%	5%	0.27 ± 0.06	0.06 (22%)
	AMD‐exploratory	72%	4%	0.31 ± 0.10	0.11 (36%)
	AMD‐structural	93%	18%	0.28 ± 0.13	0.13 (47%)
Sparse	SSE	79%	7%	0.24 ± 0.10	0.10 (42%)
	AMD‐exploratory	41%	6%	0.31 ± 0.14	0.15 (49%)
	AMD‐structural	80%	20%	0.24 ± 0.15	0.14 (60%)

^a^ The evaluation of the parameter estimates included only models with TH_CLpreg_, In AMD‐exploratory, this includes only models with CL‐PREG at a 5% significance level. In AMD‐structural, this includes all AMD‐built models, both with and without a 5% significance.

Table 3 summarizes the performance of SSE and AMD approaches for identifying the pregnancy effect on clearance (CL‐PREG) across sampling designs. Panel (A) shows the identifiability of CL‐PREG in three approaches. In SSE, the power to identify CL‐PREG was highest in the rich and medium designs and decreased with data sparsity. In AMD‐structural, power remained relatively high across all designs, while AMD‐exploratory showed a notable reduction in power, especially in sparse data scenarios. Parameter estimates for CL‐PREG (panel B) were broadly consistent across methods, although variability increased with data sparsity, especially for AMD approaches. Full LRT results and parametric power estimation (PPE) curves are available in the supporting Information (Table S1, Figure S1).

**FIGURE 2:**
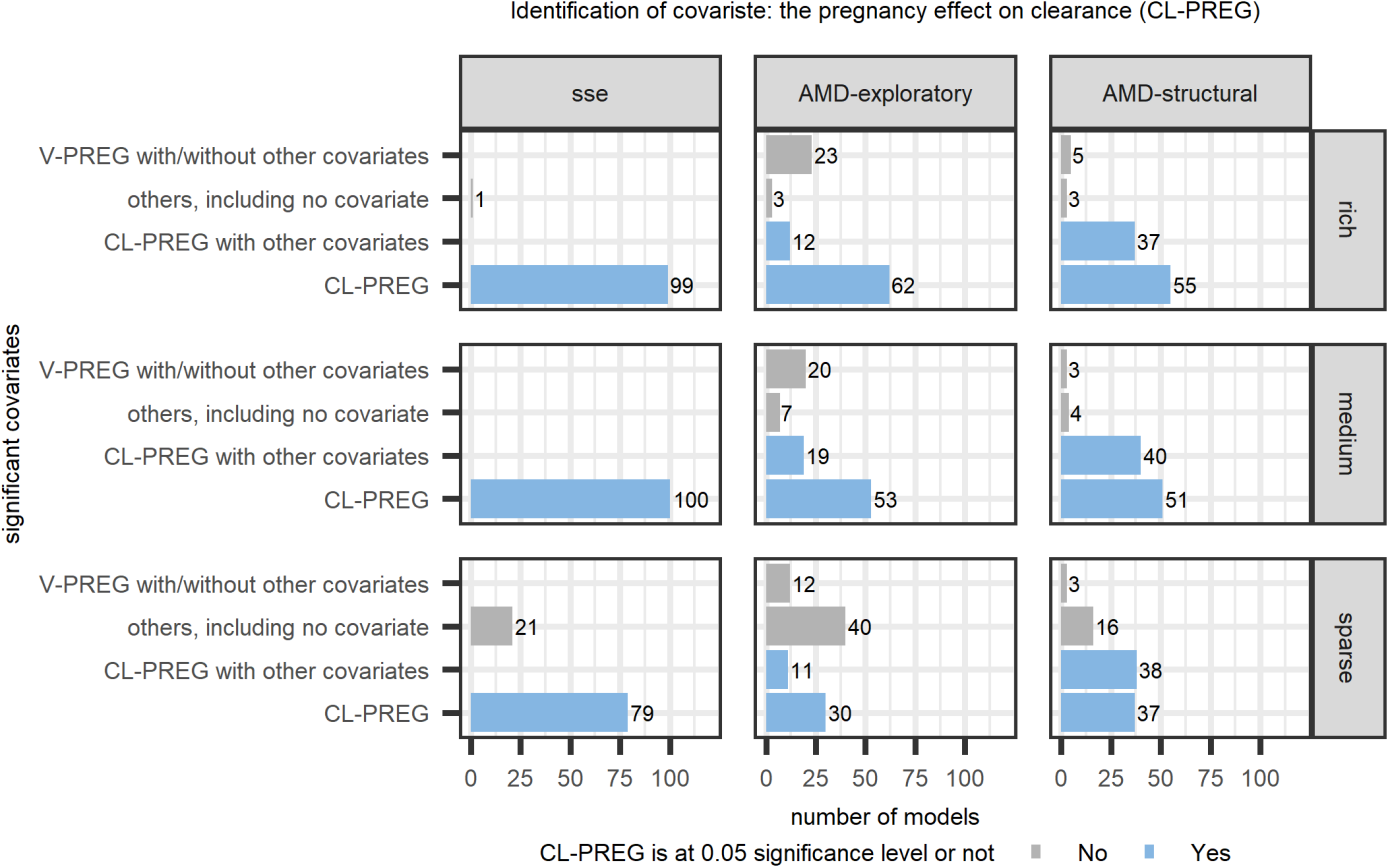
Covariate identification of three approaches: SSE, AMD‐exploratory, and AMD‐structural, using a 5% significance level and three different study designs: The rich, the medium, and the sparse design. The “others, including no covariate” category includes the pregnancy effect on mean absorption time (MAT‐PREG), age or weight effect on clearance (CL‐AGE, CL‐WT), the volume of distribution (V‐AGE, V‐WT), and the mean absorption time (MAT‐AGE, MAT‐WT).

Additionally, Figure 2 shows the significant covariates identified in each model, at a 5% significance level. In AMD‐exploratory, CL‐PREG was sometimes replaced by V‐PREG, especially in rich and medium datasets. With sparse data, most models lacked covariates, followed by V‐PREG being the second most common covariate selection. The wide covariate diversity in sparse designs suggested a limited ability to distinguish the covariate effect.

### 3.2 Bias, Precision, and Accuracy of Coefficient Estimates

Across all designs, the mean estimates of TH_CLpreg_ were generally close to or slightly overestimating the true effect of 0.25 (Table 3). AMD‐exploratory exhibited the highest bias, with a mean TH_CLpreg_ of 0.28 in rich design and 0.31 in medium to sparse designs. Precision, measured by the SD of TH_CLpreg_, was lowest in AMD‐structural followed by AMD‐exploratory and then SSE. RMSE and rRMSE of TH_CLpreg_ increased with sparser sampling and with AMD model building, as expected. Among the three approaches, AMD‐structural consistently showed the highest estimation error across all sampling designs. AMD‐exploratory yielded intermediate accuracy, while SSE provided the most accurate estimates.

### 3.3 Selection Consistency and Performance of AMD‐Built Models

#### 3.3.1 AMD Model Selection

The AMD‐exploratory approach generated 100%, 99%, and 93% successfully converged models for the rich, medium, and sparse designs, respectively, while the AMD‐structural approach successfully converged 100%, 98%, and 94% models, respectively.

The final AMD model selections are summarized in the Figures S2–S4. Structural model selection was overall consistent, while more heterogeneity was present in the selected IIV/IOV structure. The AMD tool consistently selected a one‐compartment model with first‐order absorption and elimination, similar to the simulation model, across both rich and medium designs. The sparse datasets led to more variability, with selection of a different absorption model (exploratory: 20%; structural: 25%) (Figure S2).

The IOV on CL and V, with a correlation (covariance), was the most consistently selected structure in AMD‐built models. This reflected the IOV on bioavailability in the reference full model (model S1), given that the current AMD tool does not support IOV on bioavailability (Figure S3). In sparse datasets, IOV on CL alone was also common. The IIV structures showed greater variability, especially in rich and medium designs, while sparse datasets showed consistent selection among a few key structures: IIV on CL, on V, on both CL and V with correlation, or without IIV.

The greatest variability in model structures was observed with sparse data, reflecting uncertainty in model selection when information is limited. As data richness increased, model selection became more consistent, and more complex and parameter‐rich models were selected (Figure S4).

#### 3.3.2 AMD Model Performance and Approach Comparison

Table 4 summarizes the difference in dBIC between the full model used in SSE and the AMD‐built models across different sampling designs and covariate model‐building strategies (Figure S5). AMD‐structural models exhibited greater variability and worse performance than AMD‐exploratory, especially in rich designs, indicating the risk of enforcing a covariate effect that is not strongly identifiable in the dataset. In sparse datasets, the difference between the two search strategies was less pronounced, possibly because the data lacked the resolution needed to discriminate between competing models. A direct BIC comparison of the two approaches is shown in Figure S6.

**TABLE 4:** Difference in Bayesian Information Criterion (BIC) between the full model and AMD‐built models.

Design	AMD search strategy	Percentage of models with dBIC ≤ 0	dBIC = BIC_AMD_‐BIC_full_			
			Min	Median	Max	SD
Rich	Exploratory	23%	−11	5	133	22
	Structural	25%	−12	4	274	43
Medium	Exploratory	28%	−132	3	141	34
	Structural	29%	−19	3	174	36
Sparse	Exploratory	46%	−63	0.8	94	23
	Structural	46%	−13	1	94	20

^a^ Models with dBIC ≤ 0 indicate that the AMD‐built model has a lower BIC, a better fit after accounting for model complexity, than the full SSE model.

## 4 Discussion

This study compared the standard SSE method with more realistic model building approaches in terms of their power and accuracy in identifying treatment/covariate effects. As a standard method in clinical trial simulations for assisting study design, the major limitation of SSE is that it assumes an ideal scenario where no model building is performed on the real data. While SSE showed the highest power and accuracy in covariate identification, it may be overestimated compared to more realistic scenarios when the true model is not known a priori. In contrast, AMD‐based approaches generated models with comparable performances and demonstrated the impact of different covariate modeling strategies and the incorporation of real‐world complexities with popPK model building. These findings underscore the importance of considering the uncertainty introduced by model building when evaluating study designs for covariate identification, as it can significantly influence both power estimates and parameter reliability.

The use of pregnancy as a categorical covariate allowed us to ground the true effect in a published clinical model (DolPHIN‐1) and apply a within‐individual design, isolating the impact of the model‐building strategy on power and estimation accuracy, without functional form uncertainty. Continuous covariates (e.g., weight, albumin) introduced additional uncertainty from choosing their functional form—something AMD must resolve but SSE bypasses by excluding the model building process. This asymmetry would likely widen the power gap between SSE and AMD‐based approaches further, making binary covariates such as pregnancy a more conservative and interpretable comparator for isolating the methodological differences. Moreover, many important covariates in study design are categorical (e.g., fasted/fed, formulation, concomitant medications, dose levels) supporting the relevance of this choice.

Forcing a covariate at the beginning and then subjecting it to a subsequent data‐driven selection process introduces path dependency in the selection, which in turn complicates simple interpretation of the nominal 5% test level. In this study, the AMD‐structural approach showed higher power to detect the pregnancy effect compared to AMD‐exploratory, but this came at the cost of an inflated T1E rate, up to 20%. This inflation likely stems from the model‐building strategy itself, when the model selection was biased in a way that favored inclusion of the covariate effect, even when it was not supported by the data. The differences between approaches were most pronounced with a sparse sampling design. With reduced data resolution, the power of covariate identification with AMD‐exploratory deviated more from the other approaches, with increased selection of alternative covariate structures, such as V‐PREG or no covariate at all (Figure 2). These findings suggest that when data are limited, the choice of power determination approach plays an even more critical role, and SSE power estimates should be interpreted with caution, particularly when model development is part of the planned analysis.

In both SSE and the AMD‐structural approaches, mean TH_CLpreg_ values were estimated across all simulations, regardless of covariate significance, whereas for AMD‐exploratory, estimates were naturally conditional on covariate inclusion (significance at 5%). This likely contributed to the higher average TH_CLpreg_ observed in AMD‐exploratory, reflecting a known post‐selection bias [20]. Variability in parameter estimates was also greater in sparse datasets, especially in the AMD‐structural, where the covariate effect was not always identifiable. These results emphasize the trade‐off between model‐building flexibility and the stability and accuracy of covariate effect estimates, particularly under limited sampling. The similarity between the BIC results for the true model (Model S1) and the AMD‐developed models shows that the latter provide a good trade‐off between model complexity and goodness‐of‐fit. While fewer than half of the models had a better fit than the full model (dBIC ≤ 0), a large proportion of models had dBIC values near zero, indicating that model performance was generally comparable. Importantly, these results demonstrate that even without full knowledge of the data‐generating model, AMD approaches can yield models that are competitive in terms of fit. These findings highlight the ability of automated strategies to recover parsimonious models with similar explanatory power, especially in sparse and moderate sampling designs where overfitting may be less of a risk.

The Pharmpy AMD tool mimics manual model building through automation, enabling explorations which were limited by time and resources. However, there were some challenges and limitations. Firstly, some models took more time to converge during the search of IIV structure, especially from sparse datasets. This issue could be potentially improved by enabling dynamic adjustment of estimation iterations (current default: $EST MAXEVAL = 99,999 in NONMEM) or initial parameter estimates while running, which requires further investigation. Secondly, the AMD tool currently does not allow for direct setting of initial estimates for covariate coefficients. Although this did not have a large impact on the accuracy of parameter estimations in our study, a more accurate initial estimate may potentially help reduce imprecision, improve final model performance, and/or decrease runtime. Thirdly, unlike SSE, which implements the PPE algorithm to estimate power across a range of sample sizes in a straightforward and computationally efficient way, the current AMD tool lacks a standardized framework for extrapolating the required sample size to achieve a predefined power target, making its use for study planning less efficient. Developing such functionality, if possible, would be a valuable step for future implementation. It should also be recognized that the AMD framework considered here represents a specific automated workflow and does not encompass the full range of real‐world model‐building practices.

A sequential use of SSE and AMD could be a rational combination that accounts for both efficiency and realisticness in evaluating study designs. SSE alone may suffice in early planning phases, when a well‐established structural model exists and covariate effects are expected to be large and robust, providing the study designers with the smallest sample size needed. Then, model‐building‐informed strategies, such as the AMD tool, can be performed to help understand a more realistic required sample size in cases where uncertainty about the underlying popPK model exists (e.g., lacking confidence about the true model, when multiple plausible models exist, or covariate effects are subtle and sensitive to model misspecification). Given that an SSE is possible to do at a fraction of the time it takes to do an AMD‐exploratory, it is rational to first establish the lower limit of the sample size using an SSE and then select a suitable sample size above this level to understand the properties of a more realistic design. This sequential strategy could help optimize computational resource use and reduce risks of misspecified models or underpowered trials.

Moreover, allowing for the optimization of current AMD procedures by combining the model building workflows with the PPE algorithm for extended sample size estimations may further improve the efficiency in study design. This approach could support adaptive trial designs, for instance, interim analyses, where model updates can inform real‐time adjustments to sample size or allocation ratios to maintain adequate power. More realistic, adaptive, and diverse study designs should be evaluated in future work to better understand their value and applicability across a broader range of clinical trial scenarios. This would allow for a more comprehensive assessment of how key design parameters influence power and covariate detection under different model‐building approaches. Ultimately, sequential SSE and AMD provide a pragmatic framework that balances computational efficiency with modeling realism, supporting more robust study design decisions.

## 5 Conclusion

The evaluation of three approaches (SSE, AMD‐structural, and AMD‐exploratory) allowed us to assess power for covariate identification. This work highlights the impact and need of incorporating model uncertainty in power estimation for clinical trial design, particularly in settings where the true model is unknown. Accounting for model‐building uncertainty, such as variability in structural assumptions, parameter estimates, and covariate relationships, allows for more realistic and robust power predictions by reflecting the full range of plausible models that might describe the data. Additionally, combining SSE with AMD delivers a practical approach that trades off computational cost against model realism, leading to more reliable study design choices.

## Author Contributions

Y.‐H.H., B.C., N.V., T.P.C.D., and M.O.K. wrote the manuscript; Y.‐H.H., B.C., T.P.C.D., and M.O.K. designed the research; Y.‐H.H., B.C., T.P.C.D., and M.O.K. performed the research; and Y.‐H.H. and B.C. analyzed the data.

## Funding

The work was performed with resources from the project INVENTS, which has received funding from the European Union's Horizon Europe Research and Innovation programme under grant agreement 101,136,365, the Swiss State Secretariat for Education, Research and Innovation (SERI) and by the UKRI Innovative UK under their Horizon Europe Guarantee scheme. T.P.C.D. was supported by the Swedish Research Council, grant no. VR 2022–01251. B.C. acknowledges FCT for her PhD grant (2023.05151.BDANA) and ERASMUS+. The computations were enabled by resources in project [UPPMAX 2025/2–156] provided by the National Academic Infrastructure for Supercomputing in Sweden (NAISS) at UPPMAX, funded by the Swedish Research Council through grant agreement no. 2022–06725.

## Conflicts of Interest

The authors declare no conflicts of interest.

## Supporting information


**Figure S1:** Diagnostic plots and power curve plots of full model versus the reduced model. In all scenarios (A) Rich, (B) Medium and (C) Sparse dataset, the full model was superior to the reduced model.
**Figure S2:** Results of the structural model search with the AMD tool. ABSORPTION (FO): AMD default structure, one‐compartment model with first‐order absorption and first‐order elimination; ABSORPTION (ZO): one‐compartment model with zero‐order absorption and first‐order elimination; TRANSITS (3, NODEPOT); one‐compartment model with three transit compartments, first‐order absorption, and first‐order elimination; PERIPHERALS (1): two‐compartment model with first‐order absorption and first‐order elimination. The only model identified as a two‐compartment model could be explained as a one‐compartment model, as its THVC was estimated to be 0.0000389, and THVP was 8.36.
**Figure S3:** Results of the IIV/IOV search with the AMD tool. The most commonly selected IOV structure across all designs was IOV ([CL, VC]): rich and medium—exploratory: 86 times; rich and medium—structural: 70 times; sparse—exploratory: 59 times; sparse—structural: 57 times.
**Figure S4:** Number of parameters in the models identified by AMD. This indicates model complexity between different covariate searching strategies and study designs. In general, the number of parameters in the AMD‐built models increased with increasing data information.
**Figure S5:** Comparison of the Bayesian Information Criterion (BIC) between the true model and the models selected by the Automated Model Development (AMD) tool. The solid diagonal line indicates when BIC is identical in the true model and the AMD‐selected model. Figure S6. BIC Comparison between AMD‐structural and AMD‐exploratory.
**Table S1:** Stochastic Simulation and Estimation (SSE) analysis results and likelihood ratio test (LRT) for three study scenarios. The first part includes the number of cases where dOFV < 0 (#(dOFV < 0)), the non‐centrality parameter with its confidence interval (ncp [CI]), and the power of identifying pregnancy on clearance covariate effect (power). The second part includes the LR statistic and the *p*‐value of significance test.
**Table S2:** Parameter estimates result in SSE.
**Table S3:** Parameter estimations analysis from the AMD‐built model.
Model S1. Full model which includes covariate effect. This is the simulation model used for power estimation.
Model S2. Full model which removes covariate effect. This is the simulation model used for type I error evaluation.
