# Supplementary material for: Using Stochastic Simulation‐Estimation and Automated Model Development to Assess Power and Accuracy for Covariate Identification
Source: CPT Pharmacometrics Syst Pharmacol. 2026 Jul 21;15(8):e70299. doi: 10.1002/psp4.70299 (PMC13580854; doi:10.1002/psp4.70299)
Supplement: Supplementary file 1 [file PSP4-15-e70299-s001.docx]

# Supplementary Material

**Model S1. Full model which includes covariate effect. This is the simulation model used for power estimation.**

$PROBLEM DOLUTEGRAVIR IN HIV-INFECTED PREGNANT WOMEN

$INPUT ID TIME DV AMT SS II EVID OCC PREGNANCY AGE WT

$DATA simdata1.csv IGN=@

$SUBROUTINE ADVAN2 TRANS2

$PK

; Pregnancy covariates and effects

PREGNANCY_EFFECT = 1

IF (PREGNANCY.EQ.1) THEN

PREGNANCY_EFFECT = 1 + THETA(6)

ENDIF

; Typical values for fixed parameters

TVCL = THETA(1) * PREGNANCY_EFFECT

TVV = THETA(2) ; Volume of distribution (V)

TVKA = THETA(3)	; Absorption rate constant (ka)

; Between Subject Variability (BSV)

BSVCL = ETA(1) ; BSV for Clearance

BSVV = ETA(2)	; BSV for Volume

BSVKA = ETA(3) ; BSV for Absorption Rate Constant (ka)

; Between Occasion Variability (BOV) - considering pregnancy and postpartum

BOVCL = 0.

; Define occasion-specific variability

IF (PREGNANCY.EQ.1)	BOVF1 = ETA(4)

IF (PREGNANCY.EQ.0)	BOVF1 = ETA(5)

F1=EXP(BOVF1)

; Define parameters for the maternal model

CL = TVCL * EXP(BSVCL) ; Clearance

V = TVV * EXP(BSVV) ; Volume of central compartment

KA = TVKA * EXP(BSVKA) ; Absorption rate constant

$ERROR

IPRED = A(2)/V ; Predicted concentration

PROP = IPRED * THETA(4) ; Proportional error term

ADD = THETA(5) ; Additive error term for BLQ

W = SQRT(ADD**2 + PROP**2) ; Combined error term

IRES = DV - IPRED ; Residual error

IWRES = IRES / W ; Weighted residuals

Y = IPRED + W * ERR(1) ; Observed concentrations

$THETA

1.5	; 1 CL [L/h]

25	; 2 V [L]

0.75 ; 3 KA [1/h]

0.35 ; 4 PROPORTIONAL ERROR

0 FIX ; 5 ADDITIVE ERROR

(-1,.25) ; 6 PREGNANCY_EFFECT

$OMEGA BLOCK(2)

0.065	; Variance for BSV on CL/F

0.028	; Covariance for correlation of 0.49 between CL/F and Vc/F

0.05	; Variance for BSV on Vc/F

$OMEGA BLOCK(1) 0.2 ; BSV for KA

$OMEGA BLOCK(1) 0.2 ; BOV for F in pregnancy

$OMEGA BLOCK(1) SAME ; BOV for F in postpartum

$SIGMA 1 FIX; Example error model for residuals

$ESTIMATION MAXEVAL=9999 PRINT=1 METHOD=1 INTER NOABORT NSIG=3

$TABLE ID TIME DV AMT SS II EVID OCC PREGNANCY AGE WT NOPRINT ONEHEADER NOAPPEND FILE=est_simmodel_data.csv

**Model S2. Full model which removes covariate effect. This is the simulation model used for type I error evaluation.**

$PROBLEM DOLUTEGRAVIR IN HIV-INFECTED PREGNANT WOMEN

$INPUT ID TIME DV AMT SS II EVID OCC PREGNANCY AGE WT

$DATA simdata1.csv IGN=@

$SUBROUTINE ADVAN2 TRANS2

$PK

; Pregnancy covariates and effects

PREGNANCY_EFFECT = 1

IF (PREGNANCY.EQ.1) THEN

PREGNANCY_EFFECT = 1 + THETA(6)

ENDIF

; Typical values for fixed parameters

TVCL = THETA(1) * PREGNANCY_EFFECT

TVV = THETA(2) ; Volume of distribution (V)

TVKA = THETA(3)	; Absorption rate constant (ka)

; Between Subject Variability (BSV)

BSVCL = ETA(1) ; BSV for Clearance

BSVV = ETA(2)	; BSV for Volume

BSVKA = ETA(3) ; BSV for Absorption Rate Constant (ka)

; Between Occasion Variability (BOV) - considering pregnancy and postpartum

BOVCL = 0.

; Define occasion-specific variability

IF (PREGNANCY.EQ.1)	BOVF1 = ETA(4)

IF (PREGNANCY.EQ.0)	BOVF1 = ETA(5)

F1=EXP(BOVF1)

; Define parameters for the maternal model

CL = TVCL * EXP(BSVCL) ; Clearance

V = TVV * EXP(BSVV) ; Volume of central compartment

KA = TVKA * EXP(BSVKA) ; Absorption rate constant

$ERROR

IPRED = A(2)/V ; Predicted concentration

PROP = IPRED * THETA(4) ; Proportional error term

ADD = THETA(5) ; Additive error term for BLQ

W = SQRT(ADD**2 + PROP**2) ; Combined error term

IRES = DV - IPRED ; Residual error

IWRES = IRES / W ; Weighted residuals

Y = IPRED + W * ERR(1) ; Observed concentrations

$THETA

1.5	; 1 CL [L/h]

25	; 2 V [L]

0.75 ; 3 KA [1/h]

0.35 ; 4 PROPORTIONAL ERROR

0 FIX ; 5 ADDITIVE ERROR

0 FIX ; 6 PREGNANCY_EFFECT

$OMEGA BLOCK(2)

0.065	; Variance for BSV on CL/F

0.028	; Covariance for correlation of 0.49 between CL/F and Vc/F

0.05	; Variance for BSV on Vc/F

$OMEGA BLOCK(1) 0.2 ; BSV for KA

$OMEGA BLOCK(1) 0.2 ; BOV for F in pregnancy

$OMEGA BLOCK(1) SAME ; BOV for F in postpartum

$SIGMA 1 FIX; Example error model for residuals

$ESTIMATION MAXEVAL=9999 PRINT=1 METHOD=1 INTER NOABORT NSIG=3

$TABLE ID TIME DV AMT SS II EVID OCC PREGNANCY AGE WT NOPRINT ONEHEADER NOAPPEND FILE=est_simmodel_data.csv

## SSE (Stochastic simulation and estimation)

The consistently low p-values in the likelihood ratio test (LRT) of the full-reduced models demonstrated that incorporating CL-PREG significantly improved model fit across all designs, with greater effects observed in richer datasets. In the rich and medium scenario, the LR statistic was 45.855 (1.10×10⁻¹⁰) and 40.952 (p-value 1.28×10⁻⁹), respectively, providing strong evidence for rejecting the null hypothesis and supporting the enhanced model fit. In the sparse dataset, while the improvement was less pronounced, the LR statistic of 17.74 (p-value 0.00014) still indicated a significant enhancement.

**Required input:** SSE full_model.mod -alternative_models= reduced_model.mod -samples=100 -rplot= 2 -dir=preg30

**Table S1.** Stochastic Simulation and Estimation (SSE) analysis results and likelihood ratio test (LRT) for three study scenarios. The first part includes the number of cases where dOFV < 0 (#(dOFV<0)), the non-centrality parameter with its confidence interval (ncp [CI]), and the power of identifying pregnancy on clearance covariate effect (power). The second part includes the LR statistic and the p-value of significance test.

| **Design** | **#dOFV** | **#(dOFV < 0)** | **ncp [Cl]** | **Power** |
| --- | --- | --- | --- | --- |
| **Rich** | 100 | 0 | 21.7 [19.8 - 23.5] | 99.6 |
| **Medium** | 100 | 0 | 19.6 [17.9 - 21.4] | 99.3 |
| **Sparse** | 100 | 0 | 7.7 [6.7 - 8.9] | 79.4 |
| **Design** | **LR Statistic** | **p-value** | **LRT Statistic** | **p-value for LRT** |
| **Rich** | 45.855 | 1.10 × 10⁻¹⁰ | -45.855 | 1.27 × 10⁻¹¹ |
| **Medium** | 40.952 | 1.28 × 10⁻⁹ | -40.952 | 1.56 × 10⁻¹⁰ |
| **Sparse** | 17.74 | 0.0001405 | 17.74 | 0.0001405 |

**(A) Rich	(B) Medium	(C) Sparse**

**Figure S1.** Diagnostic plots and power curve plots of full model versus the reduced model. In all scenarios (A) Rich, (B) Medium and (C) Sparse dataset, the full model was superior to the reduced model.

**Table S2.** Parameter estimates result in SSE.

| **Rich** | | | | | | |
| --- | --- | --- | --- | --- | --- | --- |
| Full model (Simulation model) | | | | | | |
|  | ofv | TH_CL_ | TH_CLpreg_ | TH_VC_ | TH_KA_ | TH_RUV_ |
| mean | -171.04 | 1.533 | 0.258 | 25.424 | 0.759 | 0.352 |
| sd | 104.97 | 0.132 | 0.069 | 2.067 | 0.101 | 0.012 |
| rmse |  | 0.135 | 2.100 | 0.101 | 0.012 | 0.069 |
|  |  | OM_IIV_CL_ | OM_IIV_CL_VC_ | OM_IIV_VC_ | OM_IIV_KA_ | OM_IOV_CL_ |
| mean |  | 0.060 | 0.028 | 0.048 | 0.154 | 0.198 |
| sd |  | 0.041 | 0.038 | 0.047 | 0.087 | 0.046 |
| rmse |  | 0.041 | 0.038 | 0.047 | 0.098 | 0.046 |
| Reduced model | | | | | | |
|  | ofv | TH_CL_ | TH_CLpreg_ | TH_VC_ | TH_KA_ | TH_RUV_ |
| mean | -148.14 | 1.718 | 1 | 25.585 | 0.768 | 0.358 |
| sd | 104.05 | 0.136 | 0 | 2.087 | 0.101 | 0.012 |
| rmse |  | 0.257 | 0.75 | 2.157 | 0.103 | 0.014 |
|  |  | OM_IIV_CL_ | OM_IIV_CL_VC_ | OM_IIV_VC_ | OM_IIV_KA_ | OM_IOV_CL_ |
| mean |  | 0.051 | 0.020 | 0.039 | 0.151 | 0.221 |
| sd |  | 0.041 | 0.036 | 0.043 | 0.089 | 0.050 |
| rmse |  | 0.043 | 0.037 | 0.044 | 0.101 | 0.054 |
| **Medium** | | | | | | |
| Full model (Simulation model) | | | | | | |
|  | ofv | TH_CL_ | TH_CLpreg_ | TH_VC_ | TH_KA_ | TH_RUV_ |
| mean | -55.70 | 1.526 | 0.269 | 25.357 | 0.757 | 0.352 |
| sd | 89.46 | 0.139 | 0.063 | 2.548 | 0.104 | 0.012 |
| rmse |  | 0.140 | 0.065 | 2.560 | 0.103 | 0.012 |
|  |  | OM_IIV_CL_ | OM_IIV_CL_VC_ | OM_IIV_VC_ | OM_IIV_KA_ | OM_IOV_CL_ |
| mean |  | 0.059 | 0.027 | 0.049 | 0.164 | 0.196 |
| sd |  | 0.044 | 0.039 | 0.048 | 0.119 | 0.043 |
| rmse |  | 0.044 | 0.039 | 0.048 | 0.124 | 0.043 |
| Reduced model | | | | | | |
|  | ofv | TH_CL_ | TH_CLpreg_ | TH_VC_ | TH_KA_ | TH_RUV_ |
| mean | -35.22 | 1.717 | 1 | 25.602 | 0.766 | 0.359 |
| sd | 88.92 | 0.152 | 0 | 2.556 | 0.106 | 0.013 |
| rmse |  | 0.264 | 0.75 | 2.613 | 0.106 | 0.016 |
|  |  | OM_IIV_CL_ | OM_IIV_CL_VC_ | OM_IIV_VC_ | OM_IIV_KA_ | OM_IOV_CL_ |
| mean |  | 0.053 | 0.022 | 0.042 | 0.158 | 0.214 |
| sd |  | 0.042 | 0.037 | 0.044 | 0.116 | 0.051 |
| rmse |  | 0.043 | 0.037 | 0.045 | 0.123 | 0.052 |
| **Sparse** | | | | | | |
| Full model (Simulation model) | | | | | | |
|  | ofv | TH_CL_ | TH_CLpreg_ | TH_VC_ | TH_KA_ | TH_RUV_ |
| mean | 41.15 | 1.580 | 0.236 | 25.262 | 0.729 | 0.357 |
| sd | 43.69 | 0.156 | 0.102 | 2.631 | 0.173 | 0.024 |
| rmse | - | 0.174 | 0.102 | 2.631 | 0.174 | 0.025 |
|  |  | OM_IIV_CL_ | OM_IIV_CL_VC_ | OM_IIV_VC_ | OM_IIV_KA_ | OM_IOV_CL_ |
| mean |  | 0.077 | 0.046 | 0.057 | 0.120 | 0.184 |
| sd |  | 0.049 | 0.046 | 0.050 | 0.187 | 0.053 |
| rmse |  | 0.050 | 0.049 | 0.050 | 0.202 | 0.055 |
| Reduced model | | | | | | |
|  | ofv | TH_CL_ | TH_CLpreg_ | TH_VC_ | TH_KA_ | TH_RUV_ |
| mean | 50.02 | 1.750 | 1 | 25.592 | 0.748 | 0.365 |
| sd | 43.15 | 0.154 | 0 | 2.698 | 0.196 | 0.024 |
| rmse |  | 0.293 | 0.75 | 2.749 | 0.195 | 0.028 |
|  |  | OM_IIV_CL_ | OM_IIV_CL_VC_ | OM_IIV_VC_ | OM_IIV_KA_ | OM_IOV_CL_ |
| mean |  | 0.068 | 0.040 | 0.050 | 0.117 | 0.201 |
| sd |  | 0.047 | 0.044 | 0.048 | 0.192 | 0.054 |
| rmse |  | 0.047 | 0.046 | 0.048 | 0.208 | 0.054 |

## Automated model development (AMD) tool

**Figure S2**. Results of the structural model search with the AMD tool. ABSORPTION(FO): AMD default structure, one-compartment model with first-order absorption and first-order elimination; ABSORPTION(ZO): one-compartment model with zero-order absorption and first-order elimination; TRANSITS(3, NODEPOT); one-compartment model with three transit compartments, first-order absorption, and first-order elimination; PERIPHERALS(1): two-compartment model with first-order absorption and first-order elimination. The only model identified as a two-compartment model could be explained as a one-compartment model, as its TH_VC_ was estimated to be 0.0000389, and TH_VP_ was 8.36.

**Figure S3.** Results of the IIV/IOV search with the AMD tool. The most commonly selected IOV structure across all designs was IOV([CL,VC]): rich and medium - exploratory: 86 times; rich and medium - structural: 70 times; sparse - exploratory: 59 times; sparse - structural: 57 times.

**Figure S4.** Number of parameters in the models identified by AMD. This indicates model complexity between different covariate searching strategies and study designs. In general, the number of parameters in the AMD-built models increased with increasing data information.

**Figure S5.** Comparison of the Bayesian Information Criterion (BIC) between the true model and the models selected by the Automated Model Development (AMD) tool. The solid diagonal line indicates when BIC is identical in the true model and the AMD-selected model.

**Figure S6**. BIC Comparison between AMD-structural and AMD-exploratory.

**Table S3.** Parameter estimations analysis from the AMD-built model

| **Rich** | | | | | | |
| --- | --- | --- | --- | --- | --- | --- |
| AMD-exploratory | | | | | | |
|  | TH_CL_ | TH_CLpreg_ | TH_CLage_ | TH_CLwt_ | TH_RUV_ | OM_IIV_CL_ |
| mean | 1.518 | 0.272 | - | - | 0.125 | 0.070 |
| sd | 0.123 | 0.073 | - | - | 0.009 | 0.039 |
| CV(%) | 8.110 | 26.814 | - | - | 6.905 | 56.332 |
| rmse | 0.122 | 0.076 | - | - | 0.009 | 0.038 |
| rRMSE(%) | 8.04 | 27.94 | - | - | 7.20 | 54.29 |
| AMD-structural | | | | | | |
|  | TH_CL_ | TH_CLpreg_ | TH_CLage_ | TH_CLwt_ | TH_RUV_ | OM_IIV_CL_ |
| mean | 1.530 | 0.263 | 0.022 | - | 0.128 | 0.067 |
| sd | 0.139 | 0.099 | - | - | 0.016 | 0.036 |
| CV(%) | 9.078 | 38.299 | - | - | 12.328 | 53.990 |
| rmse | 0.138 | 0.100 | 0.044 | - | 0.016 | 0.036 |
| rRMSE(%) | 9.02 | 38.02 | 2 | - | 12.5 | 53.73 |
| **Medium** | | | | | | |
| AMD-exploratory | | | | | | |
|  | TH_CL_ | TH_CLpreg_ | TH_CLage_ | TH_CLwt_ | TH_RUV_ | OM_IIV_CL_ |
| mean | 1.521 | 0.289 | 0.021 | 0.004 | 0.126 | 0.062 |
| sd | 0.157 | 0.059 | - | 0.005 | 0.010 | 0.032 |
| CV(%) | 10.340 | 20.381 | - | 141.421 | 8.101 | 51.203 |
| rmse | 0.156 | 0.071 | 0 | 0.005 | 0.010 | 0.031 |
| rRMSE(%) | 10.26 | 24.56 | 0 | 125 | 7.94 | 50 |
| AMD-structural | | | | | | |
|  | TH_CL_ | TH_CLpreg_ | TH_CLage_ | TH_CLwt_ | TH_RUV_ | OM_IIV_CL_ |
| mean | 1.518 | 0.278 | 0.021 | 0.004 | 0.129 | 0.064 |
| sd | 0.148 | 0.095 | 0.022 | - | 0.013 | 0.037 |
| CV(%) | 9.731 | 34.287 | 108.525 | - | 10.304 | 57.724 |
| rmse | 0.147 | 0.099 | 0.025 | 0 | 0.013 | 0.036 |
| rRMSE(%) | 9.68 | 35.61 | 119.05 | 0 | 10.1 | 56.25 |
| **Sparse** | | | | | | |
| AMD-exploratory | | | | | | |
|  | TH_CL_ | TH_CLpreg_ | TH_CLage_ | TH_CLwt_ | TH_RUV_ | OM_IIV_CL_ |
| mean | 1.491 | 0.337 | - | - | 0.136 | 0.098 |
| sd | 0.163 | 0.100 | - | - | 0.030 | 0.044 |
| CV(%) | 10.913 | 29.798 | - | - | 22.082 | 44.695 |
| rmse | 0.160 | 0.131 | - | - | 0.029 | 0.042 |
| rRMSE(%) | 10.73 | 38.87 | - | - | 7.35 | 42.86 |
| AMD-structural | | | | | | |
|  | TH_CL_ | TH_CLpreg_ | TH_CLage_ | TH_CLwt_ | TH_RUV_ | OM_IIV_CL_ |
| mean | 1.570 | 0.248 | 0.024 | 0.004 | 0.142 | 0.104 |
| sd | 0.167 | 0.135 | 0.028 | 0.001 | 0.027 | 0.056 |
| CV(%) | 10.666 | 54.388 | 115.771 | 22.262 | 19.362 | 54.188 |
| rmse | 0.167 | 0.134 | 0.034 | 0.001 | 0.027 | 0.056 |
| rRMSE(%) | 10.63 | 54.03 | 141.66 | 25 | 19.01 | 53.85 |
